# Rectal gas-induced susceptibility artefacts on prostate diffusion-weighted MRI with epi read-out at 3.0 T: does a preparatory micro-enema improve image quality?

**DOI:** 10.1007/s00261-020-02600-9

**Published:** 2020-06-04

**Authors:** Verena Plodeck, Christoph Georg Radosa, Hans-Martin Hübner, Christian Baldus, Angelika Borkowetz, Christian Thomas, Jens-Peter Kühn, Michael Laniado, Ralf-Thorsten Hoffmann, Ivan Platzek

**Affiliations:** 1 Institut und Poliklinik für Diagnostische und Interventionelle Radiologie, Universitätsklinikum Carl Gustav Carus Dresden, Fetscherstrasse 74, 01307 Dresden, Deutschland; 2 Klinik und Poliklinik für Urologie, Universitätsklinikum Carl Gustav Carus Dresden, Fetscherstrasse 74, 01307 Dresden, Deutschland

**Keywords:** Multiparametric magnetic resonance imaging, Prostate cancer, Diffusion weighted, Enema, Artefacts

## Abstract

**Purpose:**

To assess whether the application of a preparatory micro-enema reduces gas-induced susceptibility artefacts on diffusion-weighted MRI of the prostate.

**Methods:**

114 consecutive patients who received multiparametric 3 T MRI of the prostate at our institution were retrospectively enrolled. 63 patients self-administered a preparatory micro-enema prior to imaging, and 51 patients underwent MRI without bowel preparation. Two blinded readers independently reviewed the diffusion-weighted sequences regarding gas-induced artefacts. The presence/severity of artefacts was scored ranging from 0 (no artefact) to 3 (severe artefact). A score ≥ 2 was considered a clinically relevant artefact. Maximum rectal width at the level of the prostate was correlated with the administration of a micro-enema. Scores were compared between the scans performed with and without bowel preparation using univariable and multivariable logistic regression, taking into account potential confounding factors (age and prostate volume).

**Results:**

Significantly less artefacts were found on diffusion-weighted sequences after the administration of a micro-enema shortly prior to MR imaging. Clinically relevant artefacts were found in 10% in the patient group after enema, in 41% without enema. If present, artefacts were also significantly less severe. Mean severity score was 0.3 (enema administered) and 1.2 (no enema), and odds ratio was 0.137 (*p* < 0.0001) in univariable ordinal logistic regression. Inter-observer agreement was excellent (*κ* 0.801).

**Conclusion:**

The use of a preparatory micro-enema prior to 3 T multiparametric prostate MRI significantly reduces both the incidence and severity of gas-induced artefacts on diffusion-weighted sequences and thus improves image quality.

**Electronic supplementary material:**

The online version of this article (10.1007/s00261-020-02600-9) contains supplementary material, which is available to authorized users.

## Introduction

Prostate cancer is the second most common cancer worldwide and the fifth most common cause of death due to cancer among men, with a cumulative life risk of 3.73% [1]. The standard diagnostic work-up for prostate cancer is based on clinical examination including measurement of serum prostate-specific antigen (PSA) and digital rectal examination (DRE) [2]. Multiparametric magnetic resonance imaging (mpMRI) of the prostate combines anatomical and functional imaging and has become increasingly important in the diagnosis and treatment planning of prostate cancer. Its use in various settings has been discussed and recommended in several prostate cancer guidelines worldwide [2–5]. The Prostate Imaging Reporting and Data System, PIRADS v2.1, requires mpMRI, including high-resolution T2-weighted, diffusion-weighted and dynamic contrast-enhanced T1-weighted sequences at field strengths of at least 1.5 T [6].

High-resolution T2-weighted sequences have long been the mainstay of prostate imaging, but there is convincing evidence that the addition of diffusion-weighted imaging (DWI) leads to greatly improved diagnostic performance [7–9]. DWI is now an integral part of prostate MRI and shows promising results as a possible tool for risk stratification. Several studies have postulated that DWI might be useful to distinguish between low-risk lesions, which could be managed by active surveillance, and high-risk lesions, requiring a more aggressive therapeutic approach [10–15]. Moreover, DWI could also be useful as a tool to assess treatment response, e.g. to androgen-deprivation therapy, targeted photodynamic therapy or radiotherapy, as well as in the detection of recurrence [16–21]. Dynamic contrast-enhanced T1-weighted sequences, however, have recently been described as less essential for MRI of the prostate, and shorter, biparametric protocols consisting of T2-weighted and diffusion-weighted sequences have been proposed [22–27].

DWI, especially commonly used single-shot echo-planar sequences, is prone to susceptibility-related artefacts which occur at the interfaces of structures with different magnetic susceptibilities, such as air and soft tissue. These artefacts, which cause magnetic field inhomogeneities with image distortion and/or signal dropout, tend to be more pronounced at 3 T compared to lower field strengths [28–30]. During mpMRI of the prostate, air in the rectum may significantly reduce image quality of DWI due to pronounced distortion artefacts [31, 32].

Preparatory enemas to overcome bowel gas-related susceptibility artefacts on DWI of the rectum and prostate have been discussed in the literature. However, published data are conflicting, with some publications stating no benefit, others claiming improved image quality after the administration of a micro-enema [33–35]. As a result, PIRADS v2.1 discusses the implementation of bowel preparation, stating that, so far, no consensus regarding patient preparation has been reached. A preparatory enema might be helpful, but there are concerns regarding increased bowel motility and consequent motion artefacts [6].

Thus, the aim of our study was to assess the use of a preparatory micro-enema prior to multiparametric prostate MRI.

## Material and methods

### Patients

The study was approved by the local ethics committee. Due to the retrospective nature of this study, informed consent was waived.

MRIs and clinical data of 126 consecutive patients who had received mpMRI of the prostate at our institution between January 2017 and July 2017 were retrospectively reviewed. In March 2017, we had implemented a new protocol for mpMRI of the prostate including bowel preparation. This was done in the hopes to reduce the occurrence of susceptibility artefacts on DWI and after hearing promising results in regards to bowel preparation at 3 T MRI of the rectum [36].

To avoid bias, all repeat MRIs of the same patient were excluded. Eight patients were excluded because of artefacts due to hip replacements. One patient after rectal resection, one with pronounced general motion artefacts and two patients with indwelling urinary catheters were also excluded. The remaining 114 patients were included in the study. 63 patients self-administered a preparatory micro-enema prior to imaging, 51 patients underwent MRI without bowel preparation, either because they were examined prior to the implementation of the bowel preparation protocol or because they did not consent to the application. We did not perform standardized interviews after the MRI, but patients generally tolerated the micro-enema well. Patient characteristics and indications for MRI are summarized in Tables 1 and 2.

**Table 1:** Patient characteristics

	Micro-enema	No enema
Mean age (range)	64 (30–83)	66 (53–79)
Mean prostate volume in ml (range)	45.8 (9.5–249.3)	49.7 (19.5–125.7)
Mean PSA in ng/ml (range)	7.7 (0.99–38.3)	7.8 (1.8–24.5)
Prior TURP^a^	2	2
PIRADS 3 lesions on MRI	28	20
PIRADS 4 lesions on MRI	18	15
PIRADS 5 lesions on MRI	8	8
Histology not available	5	3
Prostate carcinoma^b^	33	25
Atypical small acinar proliferation (ASAP)^b^	6	3
High-grade prostatic intraepithelial neoplasia (HGPIN)^b^	1	1
Prostatitis^b^	14	9

^a^Transurethral resection of the prostate

^b^Confirmed after biopsy

**Table 2:** Indications for MRI

	Micro-enema (*n* = 63)	No enema (*n* = 51)
Elevated PSA levels	54	40
Active surveillance	5	5
Prior to prostatectomy	3	5
Suspicious DRE	1	1

### MR imaging

Patients were asked to self-administer a micro-enema (Microlax^®^, 5 ml, Johnson & Johnson) 15–30 min prior to MRI. No dietary instructions were given to patients and no other bowel preparation or spasmolytics were administered. PIRADS v2.1 discusses the administration of spasmolytics, but there is no recommendation as to routine use due to the unclear benefit, incremental cost and potential side effects [6].

All MR images were acquired on a 3 T scanner (Siemens Verio, Siemens AG, Medical Solutions) using a phased-array surface coil and a standardized routine protocol for prostate imaging. The protocol consists of one axial T1-weighted sequence, two T2-weighted sequences (sagittal and axial), axial diffusion-weighted sequences (five b values), axial dynamic contrast-enhanced T1-weighted sequences after administration of 0.1 ml/kg Gadobutrol (Gadovist^®^, Bayer Vital GmbH) intravenously, including delayed contrast-enhanced, fat-saturated T1-weighted images about 5 min post contrast injection. Apparent diffusion coefficient maps were generated automatically. For detailed sequence parameters of the diffusion-weighted sequences see Table 3.

**Table 3:** Sequence parameters of DWI

Imaging plane	Axial
Echo time (ms)	93
Repetition time (ms)	8300
Field of view (mm)	221 × 260
In-plane resolution (mm × mm)	1.625 × 1.625 × 3
Slice thickness/gap (mm)	3/3
Phase encoding direction	Anterior–posterior
Number of signals averaged	4
Parallel imaging	iPAT mode GRAPPA
Acceleration factor	2
Flip angle	90°
Matrix	160 × 136
Fat saturation technique	SPAIR
Receiver bandwidth (Hz/voxel)	1157
b values (s/mm^2^)	0, 500, 1000, 1500, 2000

*iPAT* integrated parallel acquisition techniques, *GRAPPA* generalized autocalibrating partial parallel acquisition, *SPAIR* spectral attenuated inversion recovery

### Image interpretation

MR images were reviewed at a PACS workstation (AGFA Healthcare, Impax EE R20 XVIII SU1). Two readers (one radiologist with more than 7 years, one radiology resident with more than 2 years of experience in multiparametric prostate MRI) independently reviewed DWI b1000-images of all cases for bowel-gas-related susceptibility artefacts with a negative impact on the evaluation of the prostate. Both readers also reviewed T2-weighted sequences for motion artefacts and other exclusion criteria (hip replacement, rectal resection, indwelling urinary catheter). The readers were blinded to the date of the MRI, medical history, bowel preparation and referral diagnoses.

**Fig. 1:**
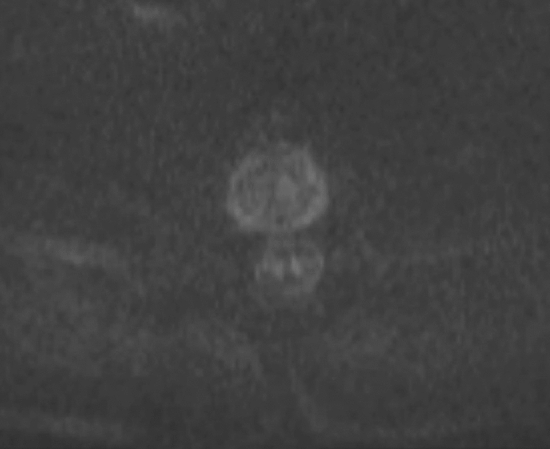
Artefact score 0 (b1000)

**Fig. 2:**
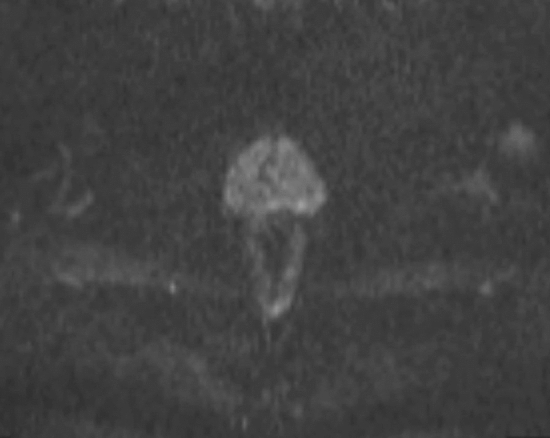
Artefact score 1 (b1000)

**Fig. 3:**
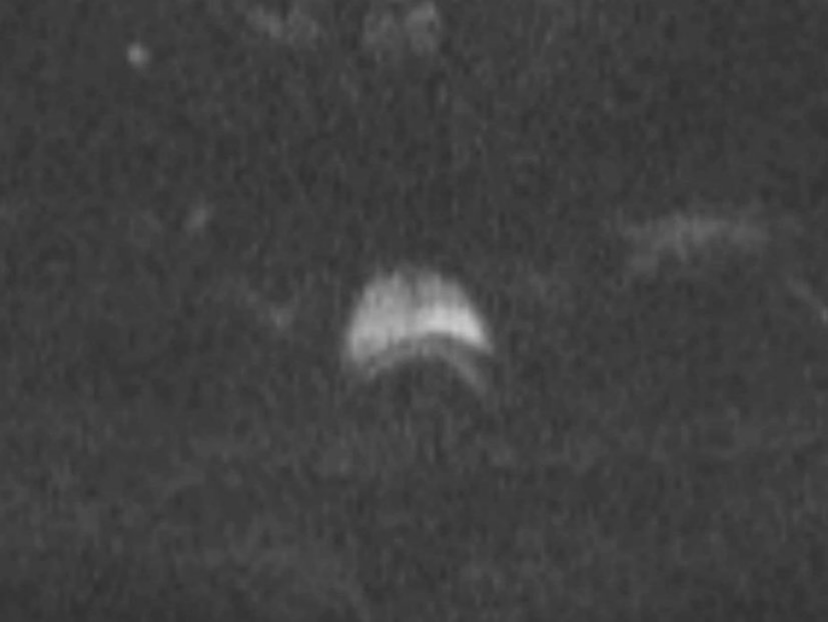
Artefact score 2 (b1000)

**Fig. 4:**
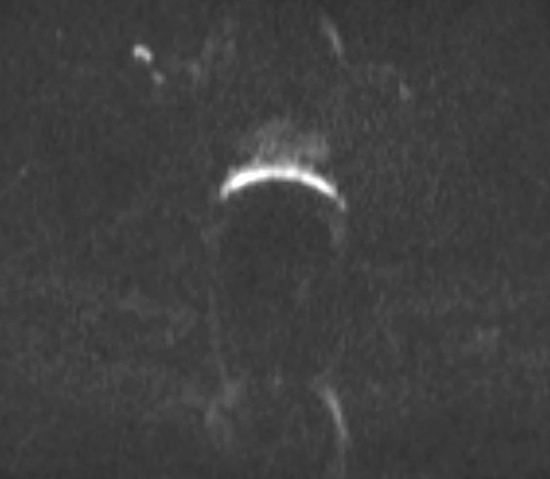
Artefact score 3 (b1000)

Artefacts were scored from 0 to 3 (0 = no artefacts, 1 = mild artefacts, 2 = moderate artefacts, 3 = severe artefacts). No artefact was defined as good delineation of prostate contours and no image distortion of the prostate, mild artefact as mild distortion, with the bigger part of the prostate still amenable to imaging analysis. Moderate artefact was defined as marked distortion and/or blur concerning the bigger part of the prostate, with some remaining areas still assessable, severe artefact as severe distortion with severe signal pile-up, leading to non-diagnostic images (see Figs. 1, 2, 3, 4). Prior to reviewing patient scans, both readers were trained using exemplary cases to ensure consistent artefact scoring. The maximum width (measured on the sagittal T2-weighted sequence) of the rectum at the level of the prostate was also documented. Prostate volume was calculated using the following formula: Length × Width × Height × *π*/6. Prostate volume and maximum rectal width were determined by both readers in consensus.

### Analysis of clinical data

Patient files were reviewed to determine patient age and indication for MRI.

### Statistical analysis

We used descriptive statistics and the Shapiro–Wilk test to assess the normal distribution for numerical variables. As the data were not normally distributed, we used the Mann–Whitney *U* test to assess differences between both groups (enema versus no-enema group). For the purpose of statistical analysis, artefact scores of ≤ 1 (no/mild artefacts) and ≥ 2 (moderate/severe artefacts) were pooled and an artefact score ≥ 2 was defined as clinically relevant artefacts. The proportion of clinically relevant artefacts in the no micro-enema versus micro-enema group was assessed as a primary outcome, the artefact severity score itself as a secondary outcome. Univariate analysis using Mann–Whitney *U* test was performed to assess the differences between both groups regarding rectal width. Univariable and multivariable logistic regression was used to assess potential confounding factors (age and prostate volume), with a binary outcome for the primary outcome and ordinal logistic regression for the secondary outcome. *p* < 0.05 was considered to indicate statistically significant differences. Inter-observer agreement was assessed using Cohen’s Kappa (0–0.2 = poor, 0.21–0.4 = fair, 0.41–0.6 = moderate, 0.61–0.8 = good and 0.81–1.00 = excellent agreement).

Statistical analysis was performed using SPSS package for Windows, SPSS Statistics 23, IBM.

## Results

Mean age of all patients was 65 years (30–83 years). Mean prostate volume measured 58.3 ml (9.5–249.3 ml). Age and prostate volume were not statistically different between both groups of patients (no enema/enema, *p* ≥ 0.05). In both groups, there were two patients after TURP.

**Fig. 5:**
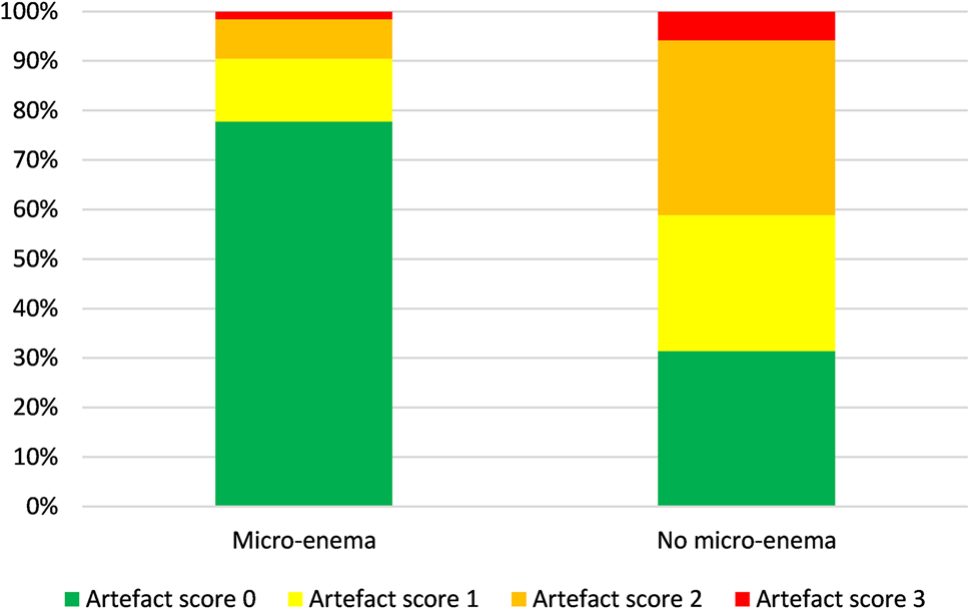
Artefact scores in the enema versus no-enema group

Diffusion-weighted sequences on MRI of patients in the group after self-administration of micro-enema showed significantly less clinically relevant (≥ 2) artefacts compared to the no-enema group, with an odds ratio of 0.150 (*p* = 0.0002). Clinically significant artefacts were present in 10% in the enema group and in 41% in the no-enema group. Artefacts were also significantly less severe in the enema group, with an odds ratio of 0.137 (*p* < 0.0001). The mean severity score in the enema group was 0.3 versus 1.2 in the no-enema group (see Table 4, Fig. 5). Rectal width significantly reduced after enema in univariate analysis. Maximum width of the rectal ampulla was 2.56 cm in the enema group, compared to 3.19 cm in the no-enema group.

**Table 4:** Results for MRI scans with and without bowel preparation

	Micro-enema (*n* = 63)	No enema (*n* = 51)
Relevant artefacts (≥ 2)		
Yes	6 (9.5%)	21 (41.2%)
No	57 (90.5%)	30 (58.8%)
Artefact scores		
0	49 (77.8%)	16 (31.4%)
1	8 (12.7%)	14 (27.4%)
2	5 (7.9%)	18 (35.3%)
3	1 (1.6%)	3 (5.9%)

Inter-observer agreement was excellent (*κ* 0.801, 95% confidence interval 0.69–0.88).

## Discussion

Our study demonstrates that bowel preparation using a self-administered micro-enema is feasible and leads to significantly fewer, as well as less severe artefacts on DWI sequences of the prostate. The additional cost of bowel preparation in this setting is low (ca. 1 € per micro-enema) and, although we did not perform standardized interviews, patients generally tolerated the micro-enema well.

Prior studies have reported conflicting results, with one study by Lim et al. finding no significant reduction of artefacts or improvement of image quality after the use of a preparatory enema prior to mpMRI of the prostate at 3 T [33]. This retrospective study included 60 patients who were asked to administer a cleansing enema (Fleet enema, C.B. Fleet) the morning of the scheduled MRI. Several factors might have led to the results, which are contrary to our findings. The authors do not provide data on the time lag between administration of the enema and MRI, but relatively long delays may have had a negative impact on artefact reduction. Rectal stool and gas in the study by Lim et al. were both scored on a five-point scale (no rectal stool/gas to large amount of rectal stool/gas), based on subjective assessment, which could introduce bias into the findings. In our study, we also subjectively assessed and scored artefacts, but included measurements of the maximum rectal width at the level of the prostate. Maximum rectal width, depending on the amount of stool/air in the rectal ampulla, was significantly lower after the administration of a micro-enema (*p* < 0.001). Only 16% of patients in the no-enema group were reported by Lim et al. as showing moderate to large amounts of stool and no patient in either group was recorded as having moderate to large amounts of rectal gas, which could explain why a preparatory enema did not lead to a significant reduction in artefacts. Also, the number of patients included was relatively low (with 28 patients having received bowel preparation).

Another recent study retrospectively evaluated 117 patients under active surveillance who underwent prostate MRI both without and with bowel preparation obtained within 12 months of each other [34]. In this study, fleet’s enema was administered about 12 h prior to the MRI scan. Two readers (one radiologist, one urologist) reviewed images regarding rectal distension, rectal anterior–posterior (AP) diameter, DWI distortion and DWI artefacts. DWI distortion and rectal AP diameter were assessed quantitatively, whereas rectal distension and DWI artefacts were assessed using scoring systems. The results are partially in keeping with our findings, insofar as rectal diameter was significantly lower in the patient group with bowel preparation. The radiologist reader also found rectal distension on a subjective Likert scale to be significantly lower in this group, whereas for the urologist reader this was not significant.

Regarding DWI assessment, only the radiologist reader found significant improvement of DWI distortion after bowel preparation, both readers did not find significant differences in DWI artefacts on qualitative scoring. Inter-observer agreement in this study by Coskun et al. was weak for DWI distortion and artefacts. A possible explanation could be that the urologist reader approached imaging and especially artefact assessment from a different point of view, presumably being primarily trained to look for malignancy. Artefact scoring was performed reviewing scans for blurring or subtle artefact lines, poor signal-to-noise ratio or prominent artefact lines, but no training was performed prior to the evaluation and no image examples demonstrating artefact scoring are provided. We tried to standardize subjective artefact scoring by training readers as a first step and by employing specific definitions of artefact scores, which led to excellent inter-observer agreement. Another interesting point is that mean rectal AP diameter without bowel preparation was markedly lower than in our study (2.67 versus 3.19 cm), whereas mean diameter after enema was similar (2.48 versus 2.56 cm). Bowel preparation is more useful, the more stool/gas is found in the rectal ampulla, which could be another explanation for the difference in results and conclusion. Lastly, including patients receiving mpMRI within a 12-month period might have led to significant changes like progressive benign prostate hyperplasia or intercurrent repeat biopsy.

A retrospective study by Griethuysen et al. included 335 MRI scans of the rectum at 1.5 T. Patients were asked to self-administer a cleansing enema shortly prior to MRI. The authors concluded that the incidence and severity of gas-induced artefacts on DWI was significantly reduced after the administration of a micro-enema [35]. Although this study examined patients who received MRI of the rectum, methodology was similar and the results support our findings.

Several MRI techniques have been reported to reduce artefacts in diffusion-weighted imaging, such as reduction of the field-of-view, the application of segmented read-out epi sequences or parallel imaging [37–40]. However, Griethuysen et al. concluded that the effect of these techniques was less than that of a preparatory micro-enema (combined with parallel imaging) [35].

Interestingly, we did not encounter an increased rate of motion artefacts after the administration of the micro-enema, which has been a concern described in the literature [6]. We did not administer spasmolytics, but had to exclude only one patient due to pronounced motion artefacts. This could be due to the fact that we used only a micro-enema (5 ml), instead of a larger enema, which might lead to more pronounced bowel motion.

There are several limitations to our study. Firstly, data were analysed retrospectively and we did not perform test–retest evaluations. Secondly, the score implemented to quantify the presence and severity of artefacts is based on subjective visual assessment, which might hamper reproducibility. We tried to counter this limitation by using a standardized scoring system, for which readers received a short training course prior to reviewing the MRI scans. As a result, excellent inter-observer agreement between the two readers was reached. Thirdly, readers were blinded to the administration of micro-enema but suggestive imaging findings such as fluid, but no stool and/or gas in the rectal ampulla might have introduced a bias as to patient preparation. Another limitation is the inter-individual study design, although patient characteristics were comparable in both groups. Since our aim was to evaluate the influence of a micro-enema on time-efficient diffusion-weighted imaging in a clinical routine setting, we kept all other imaging parameters constant. Combining the administration of a micro-enema with different imaging techniques like non-epi read-out, different excitation methods or B0 mapping could lead to further reduction of artefacts.

In conclusion, our study provides evidence that a preparatory micro-enema prior to mpMRI of the prostate is feasible and leads to improved image quality on DWI. Prospective studies are necessary to confirm our results, and the combination of advanced imaging techniques with bowel preparation should be explored further.

## Electronic supplementary material

Below is the link to the electronic supplementary material.

Electronic supplementary material 1 (PDF 1564 kb)

Electronic supplementary material 2 (XLSX 177 kb)

## Data Availability

All data and material are available from the corresponding author.

## Abbreviations

PSA: Prostate specific antigen
DRE: Digital rectal examination
TRUS: Transrectal ultrasound
mpMRI: Multiparametric MRI
EPI: Echo planar imaging
