# Correction to: Rectal gas‑induced susceptibility artefacts on prostate diffusion‑weighted MRI with epi read‑out at 3.0 T: does a preparatory micro‑enema improve image quality?

**DOI:** 10.1007/s00261-021-03157-x

**Published:** 2021-07-06

**Authors:** Verena Plodeck, Christoph Georg Radosa, Hans‑Martin Hubner, Christian Baldus, Angelika Borkowetz, Christian Thomas, Jens‑Peter Kuhn, Michael Laniado, Ralf‑Thorsten Hoffmann, Ivan Platzek

**Affiliations:** 1 Institut und Poliklinik für Diagnostische und Interventionelle Radiologie, Universitätsklinikum Carl Gustav Carus Dresden, Fetscherstrasse 74, 01307 Dresden, Deutschland; 2 Klinik und Poliklinik für Urologie, Universitätsklinikum Carl Gustav Carus Dresden, Fetscherstrasse 74, 01307 Dresden, Deutschland

## Correction to: Abdominal Radiology (2020) 45:4244–4251 10.1007/s00261-020-02600-9

The article Rectal gas-induced susceptibility artefacts on prostate diffusion-weighted MRI with epi read-out at 3.0 T: does a preparatory micro-enema improve image quality?”, written by Verena Plodeck, Christoph Georg Radosa, Hans-Martin Hubner, Christian Baldus, Angelika Borkowetz, Christian Thomas, Jens-Peter Kuhn, Michael Laniado, Ralf-Thorsten Hoffmann, Ivan Platzek, was originally published electronically on the publisher’s internet portal on 5 June 2020 without open access. With the author(s)’ decision to opt for Open Choice the copyright of the article changed on 24 May 2021 to © The Author(s) 2020 and the article is forthwith distributed under a Creative Commons Attribution 4.0 International License, which permits use, sharing, adaptation, distribution and reproduction in any medium or format, as long as you give appropriate credit to the original author(s) and the source, provide a link to the Creative Commons licence, and indicate if changes were made. The images or other third party material in this article are included in the article’s Creative Commons licence, unless indicated otherwise in a credit line to the material. If material is not included in the article’s Creative Commons licence and your intended use is not permitted by statutory regulation or exceeds the permitted use, you will need to obtain permission directly from the copyright holder. To view a copy of this licence, visit http://creativecommons.org/licenses/by/4.0.

The original article has been corrected.

