# Supplementary material for: Rectal gas-induced susceptibility artefacts on prostate diffusion-weighted MRI with epi read-out at 3.0 T: does a preparatory micro-enema improve image quality?
Source: Abdom Radiol (NY). 2020 Jun 4;45(12):4244–51. doi: 10.1007/s00261-020-02600-9 (PMC8260527; doi:10.1007/s00261-020-02600-9)
Supplement: Supplementary file 1 — Electronic supplementary material 1 (PDF 1564 kb) [file 261_2020_2600_MOESM1_ESM.pdf]

Rectal gas-induced susceptibility artefacts on prostate diffusion-weighted MRI with epi read-out at 3.0 Tesla – Does a preparatory micro-enema improve image quality?

Abdominal Radiology

Authors:

Verena Plodeck[1], Christoph Georg Radosa[1], Hans-Martin Hübner[1], Christian Baldus[1], Angelika Borkowetz[2], Christian Thomas[2], Jens-Peter Kühn[1], Michael Laniado[1], Ralf-Thorsten Hoffmann[1], Ivan Platzek[1]

[1]Institut und Poliklinik für Diagnostische und Interventionelle Radiologie, Universitätsklinikum Carl Gustav Carus Dresden, Fetscherstrasse 74, 01307 Dresden, Deutschland

[2]Klinik und Poliklinik für Urologie, Universitätsklinikum Carl Gustav Carus Dresden, Fetscherstrasse 74, 01307 Dresden, Deutschland

Corresponding author:

Verena Plodeck

Institut und Poliklinik für Diagnostische und Interventionelle Radiologie, Universitätsklinikum Carl Gustav Carus Dresden, Fetscherstrasse 74, 01307 Dresden, Deutschland

Telephone: 0049 351 458 18405

Fax: 0049 351 458 4321

Email address: verena.plodeck@uniklinikum-dresden.de

<u>Training cases</u>

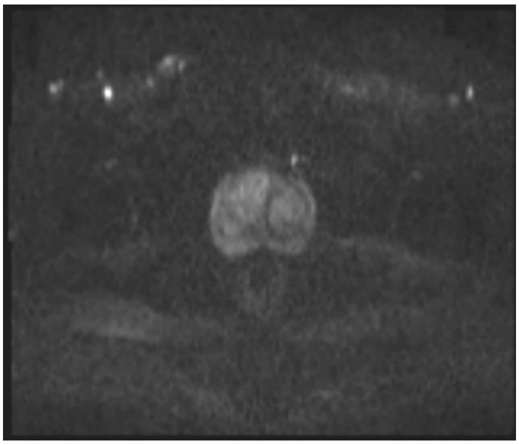

Case 1 Artefact score 0

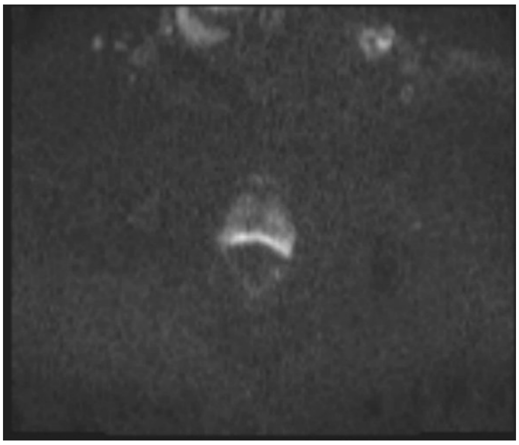

Case 2 Artefact score 1

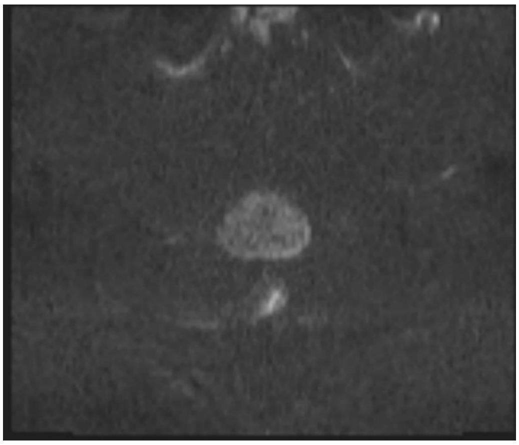

Case 3 Artefact score 0

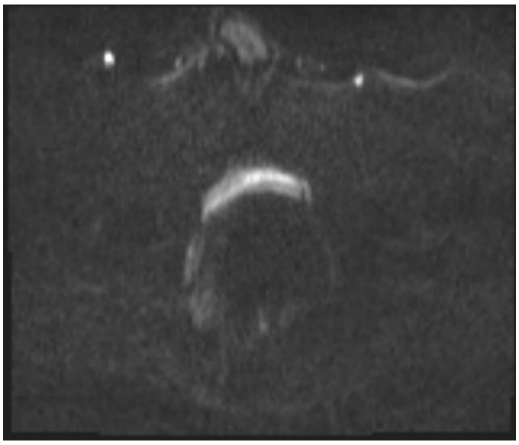

Case 4 Artefact score 3

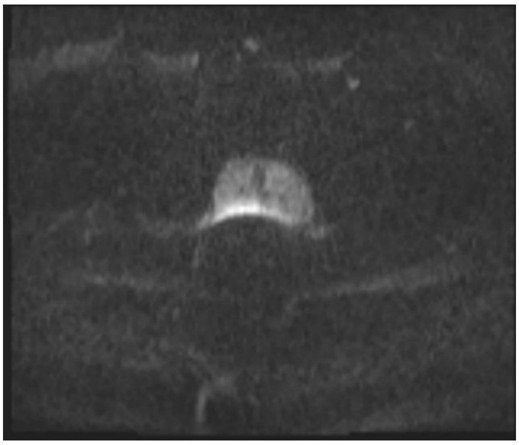

Case 5 Artefact score 1

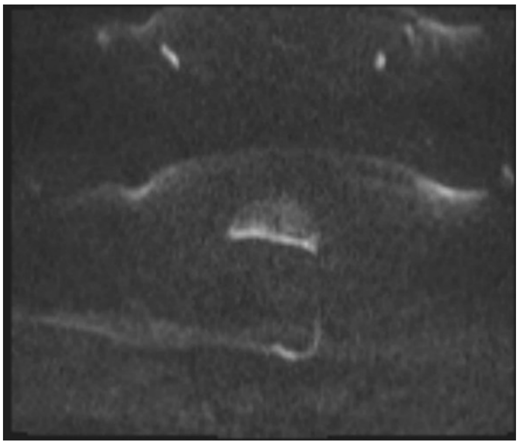

Case 6 Artefact score 2

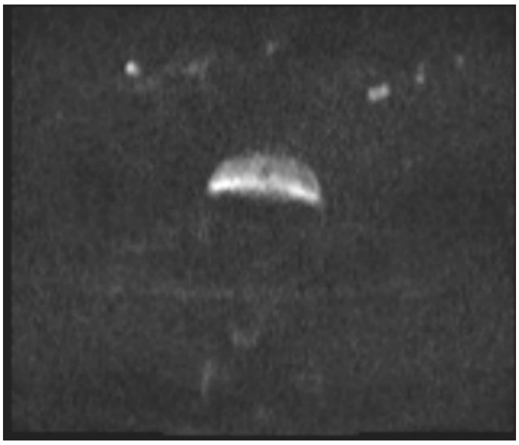

Case 7 Artefact score 2

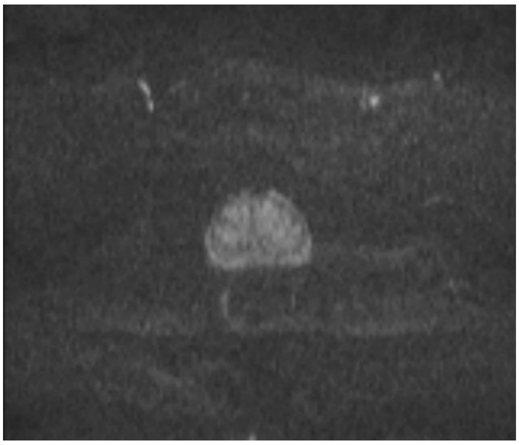

Case 8 Artefact score 0

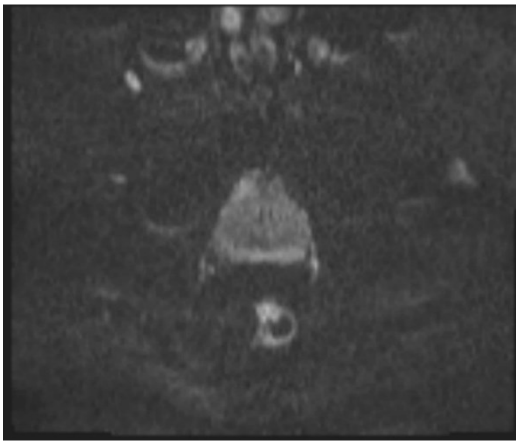

Case 9 Artefact score 0

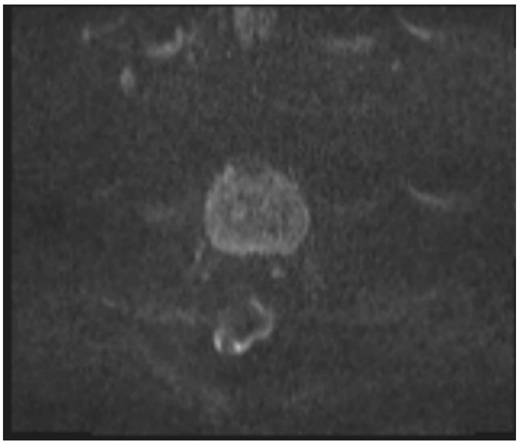

Case 10 Artefact score 0

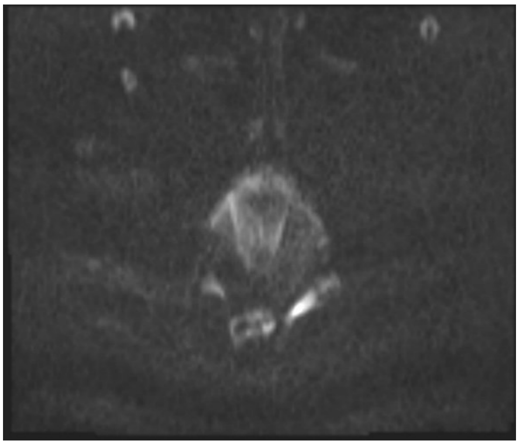

Case 11 Artefact score 3

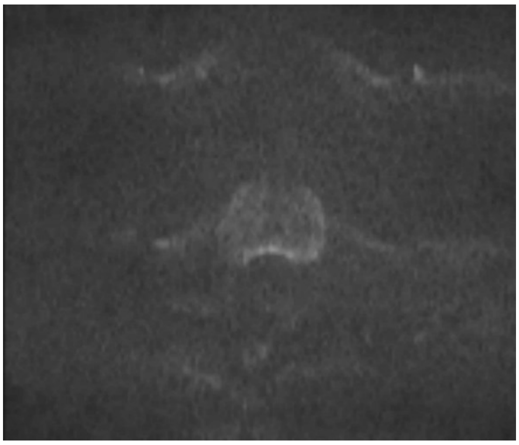
Case 12 Artefact score 1

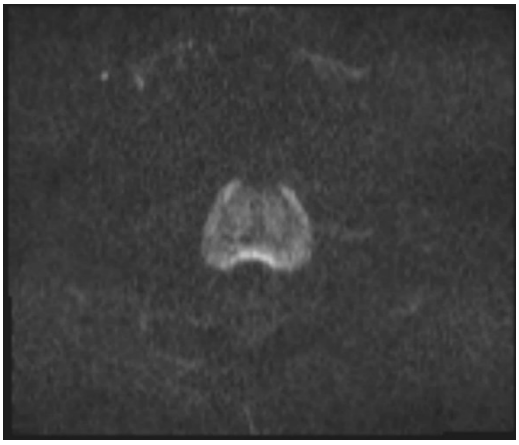
Case 13 Artefact score 1

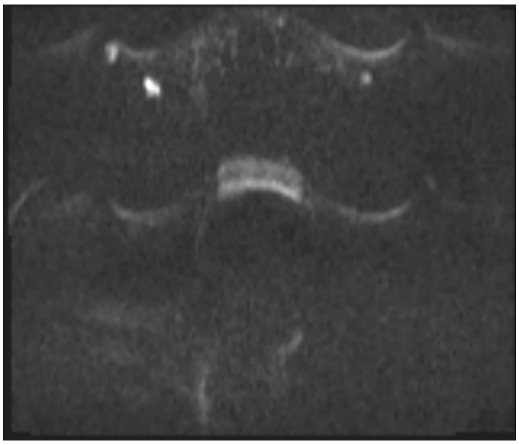
Case 14 Artefact score 3

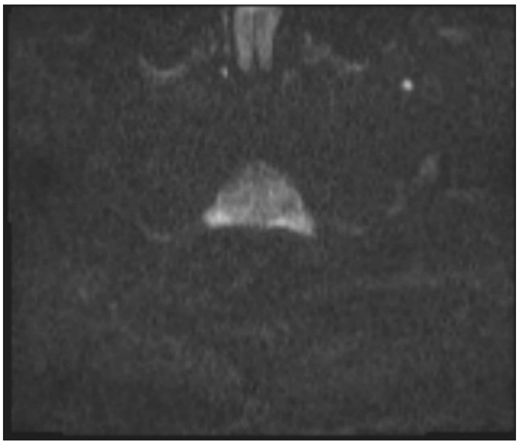
Case 15 Artefact score 1

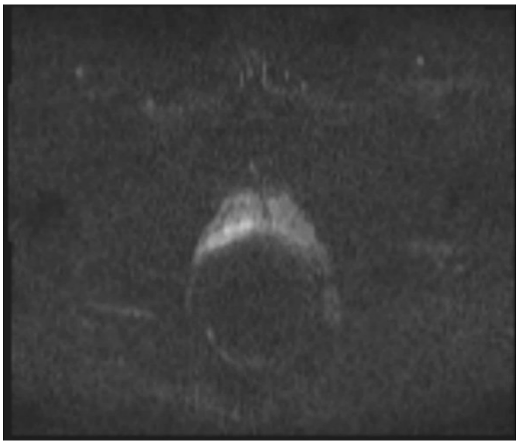
Case 16 Artefact score 2

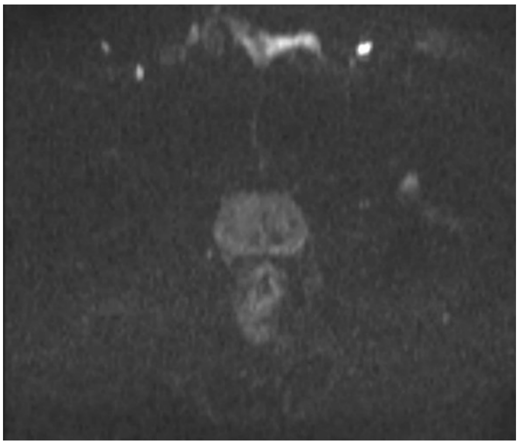
Case 17 Artefact score 0

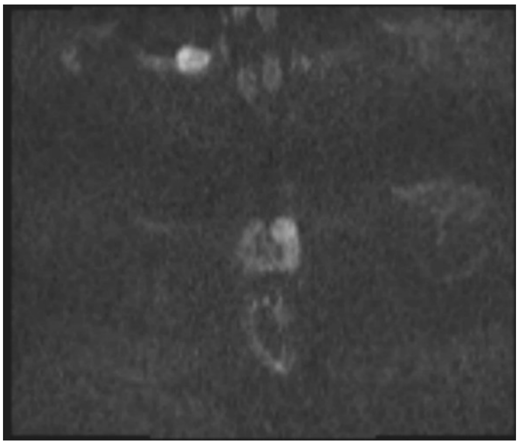
Case 18 Artefact score 0

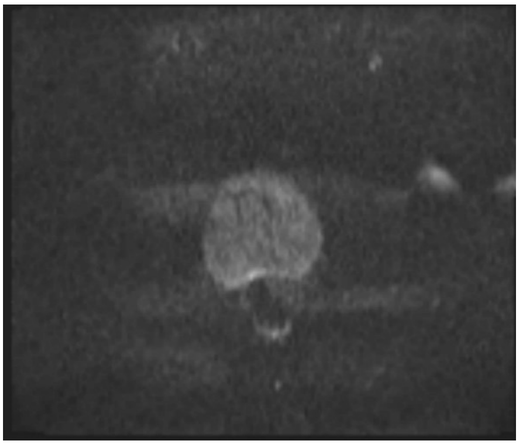
Case 19 Artefact score 1

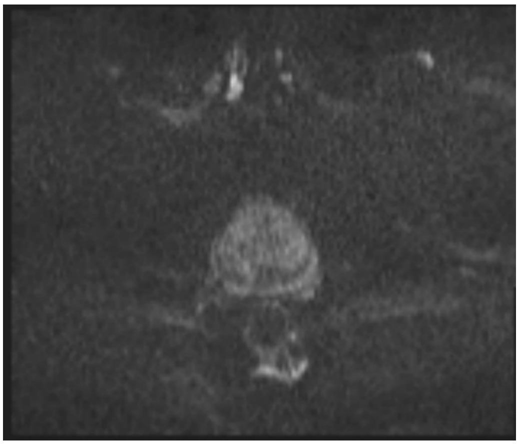

Case 20 Artefact score 0

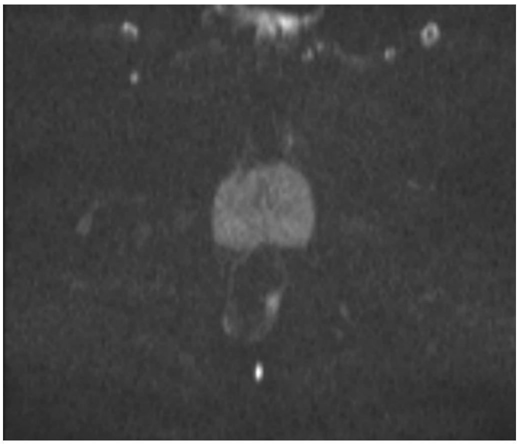

Case 21 Artefact score 0

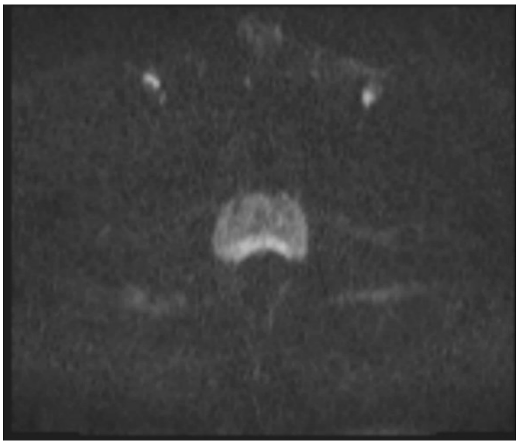

Case 22 Artefact score 1

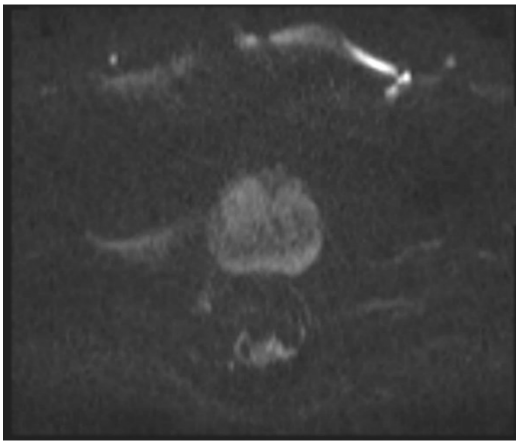

Case 23 Artefact score 0

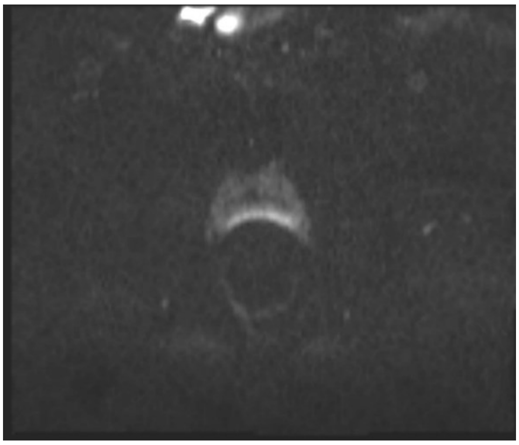

Case 24 Artefact score 1

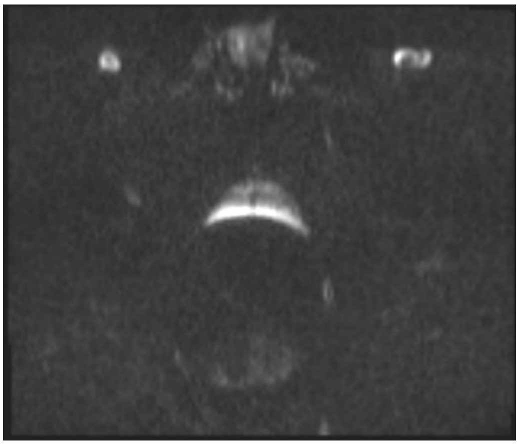

Case 25 Artefact score 2

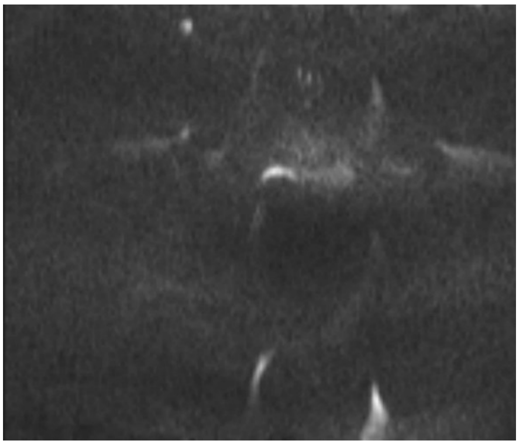

Case 26 Artefact score 3

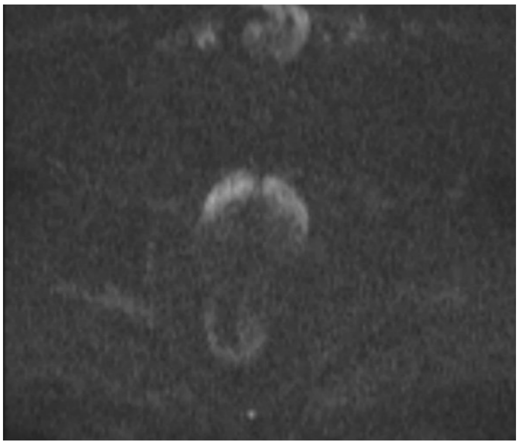

Case 27 Artefact score 2

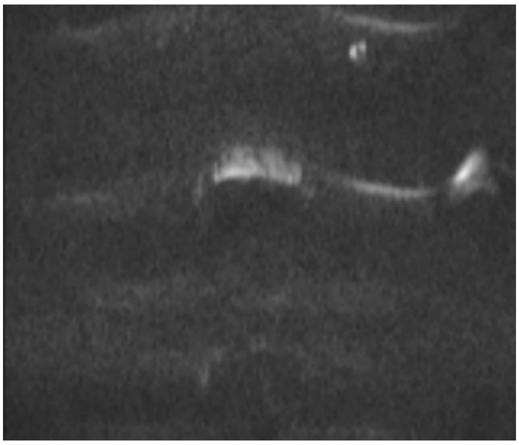

Case 28 Artefact score 2

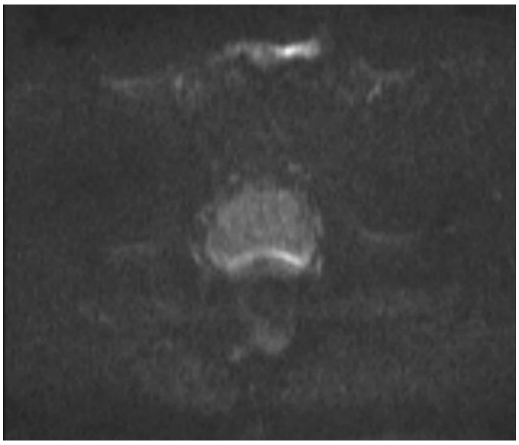

Case 29 Artefact score 1

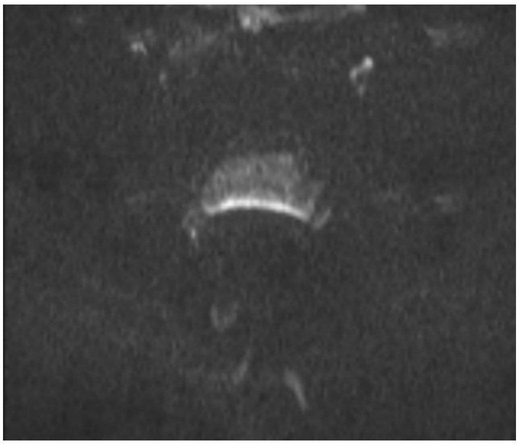

Case 30 Artefact score 2

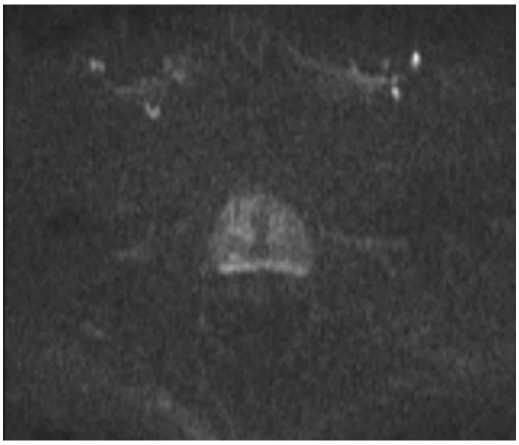

Case 31 Artefact score 1

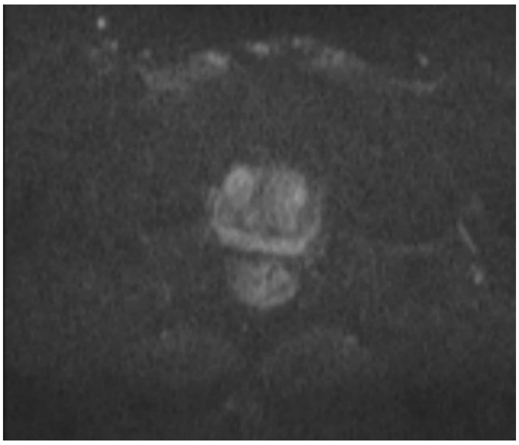

Case 32 Artefact score 0

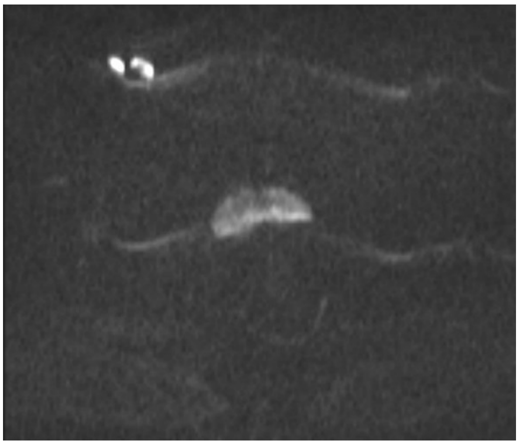

Case 33 Artefact score 2

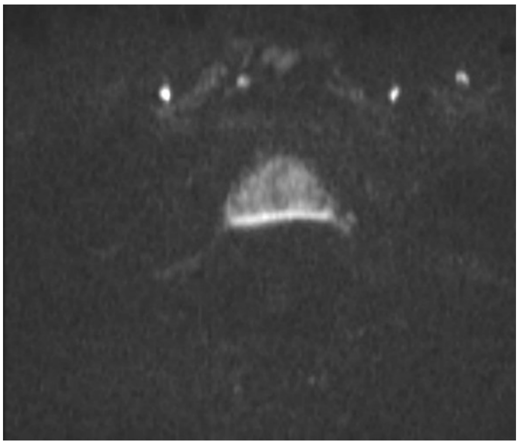

Case 34 Artefact score 1

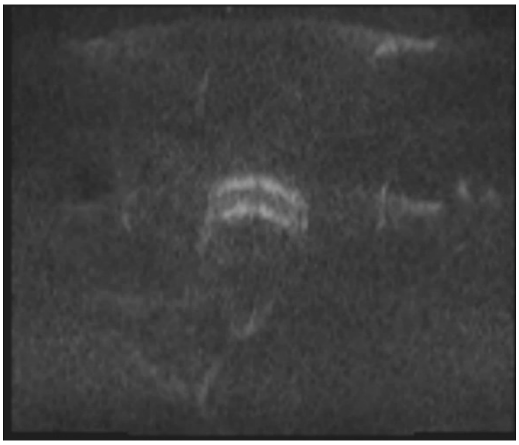

Case 35 Artefact score 3

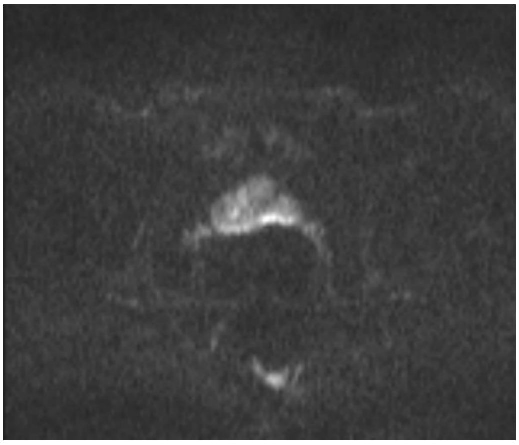

Case 36 Artefact score 2

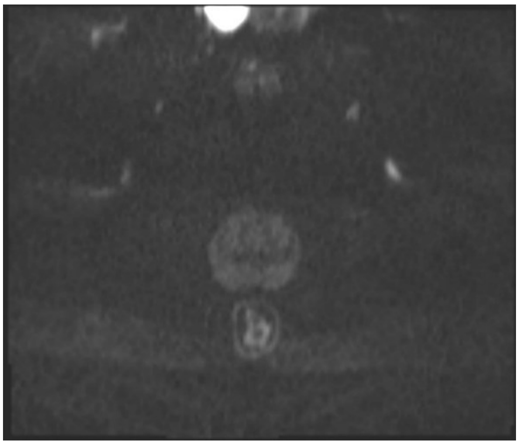

Case 37 Artefact score 0

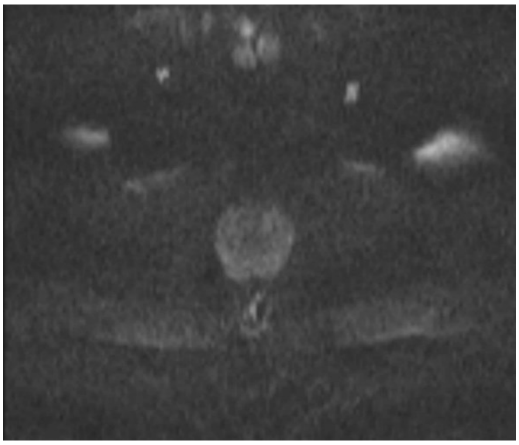

Case 38 Artefact score 0

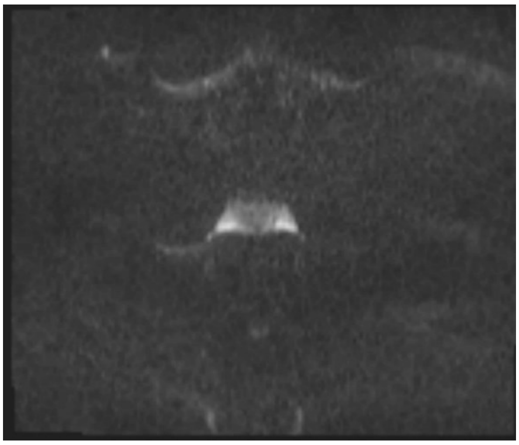

Case 39 Artefact score 2

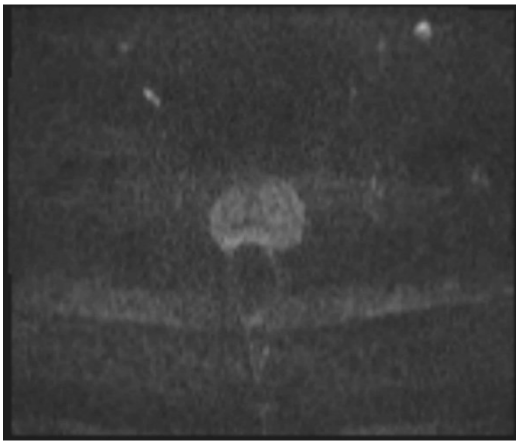

Case 40 Artefact score 1

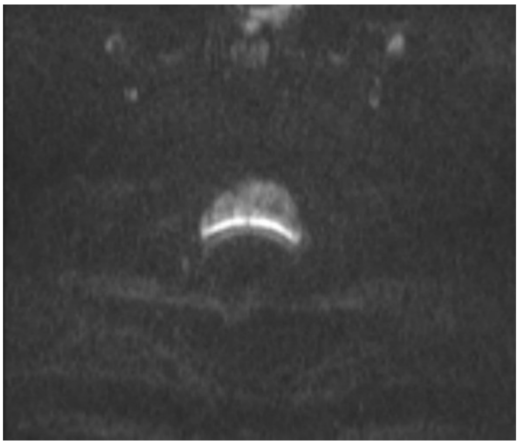

Case 41 Artefact score 2

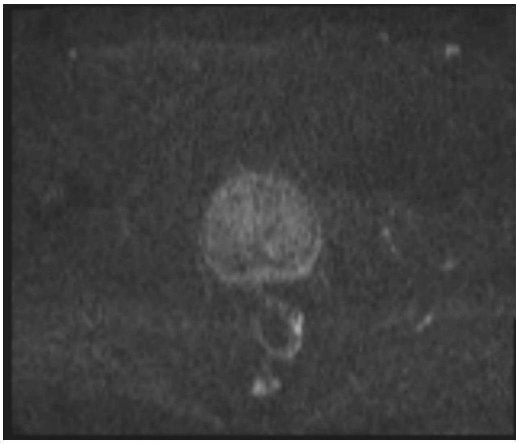

Case 42 Artefact score 0

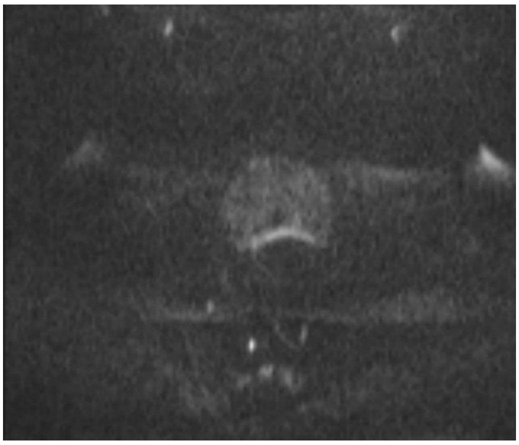

Case 43 Artefact score 1

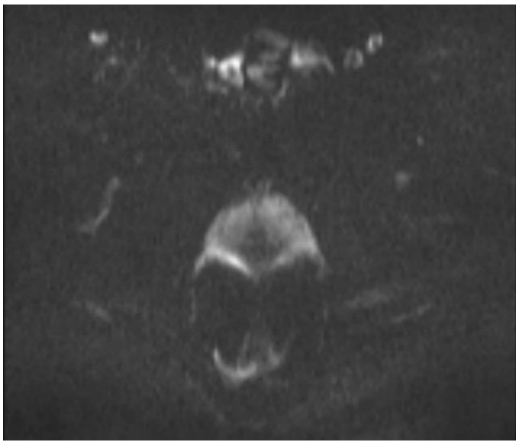

Case 44 Artefact score 2

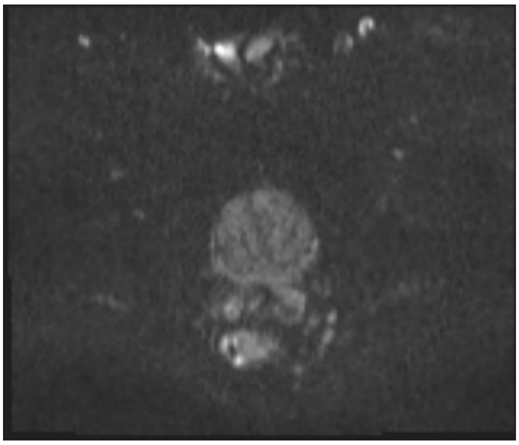

Case 45 Artefact score 0

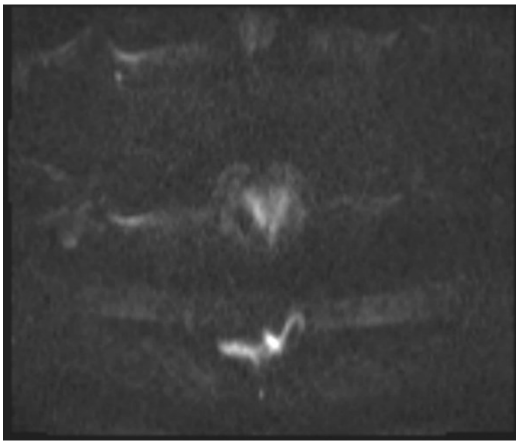

Case 46 Artefact score 3

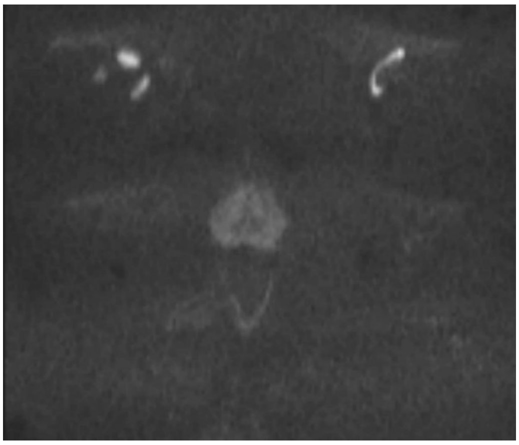

Case 47 Artefact score 0

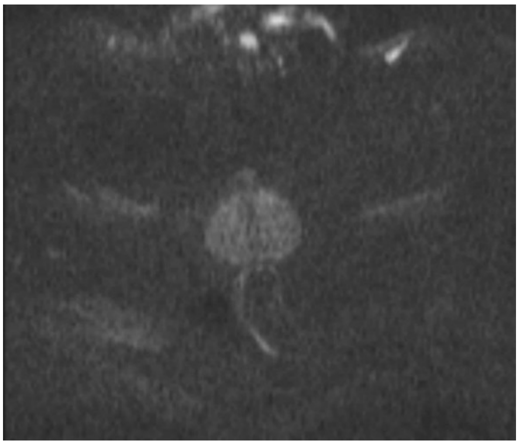

Case 48 Artefact score 0

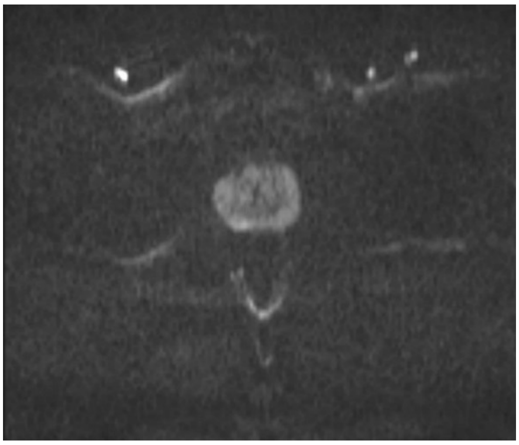

Case 49 Artefact score 0

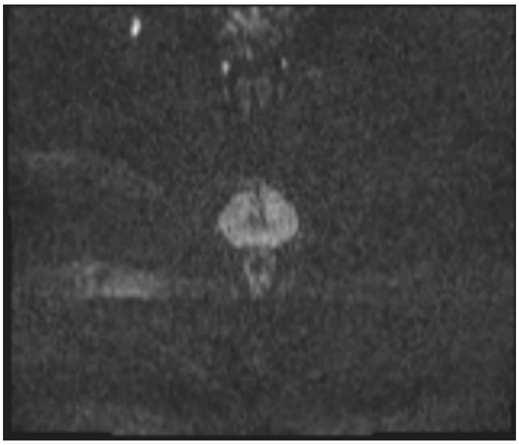

Case 50 Artefact score 0
